# Cross-cultural differences in social and self-referential memory are magnified with age

**DOI:** 10.1080/13825585.2026.2691113

**Published:** 2026-06-20

**Authors:** Iva Dujmic, Isu Cho, Angela Gutchess, Yu-Ling Chang

**Affiliations:** a Department of Psychology, Brandeis University, Waltham, MA, USA;; b Department of Psychology, Sungkyunkwan University, Seoul, South Korea;; c Department of Psychology, National Taiwan University, Taipei, Taiwan

**Keywords:** Aging, culture, memory, self-referencing, social cognition

## Abstract

Social content and thinking about information in relation to the self may attenuate age-related declines in associative memory. However, it’s unclear whether these strategies are similarly effective across cultures due to potential differences in the prioritization of social and self-relevant information. The present study compared Taiwanese and American younger and older adults on associative memory for social information across two encoding conditions. Specifically, they related object-scene image pairs containing varying levels of social information (i.e. none, low and high) to themselves (i.e. self-referencing) or to a distant-other (i.e. other-referencing). We replicated a prior finding in a new sample of Taiwanese participants in which other-referencing (vs self-referencing) enhanced older adults’ memory for high social trials, relative to younger adults. In contrast, this pattern did not emerge for Americans; older American participants’ memory performance was relatively consistent across self- vs. other-referencing and the level of social information, and there was no interaction with age. These findings suggest that cultural differences in memory for high social (relative to low and nonsocial) information emerge for older, but not younger, adults, particularly when participants are asked to think about another person. Therefore, culture may influence the effectiveness of strategies to reduce age-related associative memory impairments.

Identifying ways to support memory with age is an important endeavor, and increasingly, there is interest in understanding individual differences in the types of information or strategies that are most effective for different people (e.g., Leal et al., 2025). Culture is one source of individual differences and the ways in which it can modify trajectories of age-related decline in memory are beginning to be understood (Gutchess & Cho, 2024), with some research across cultures on associative memory (e.g., Chen & Chang, 2016; Yang et al., 2013). Associative memory, the ability to bind pieces of information together, is particularly impaired with age (Naveh-Benjamin, 2000; Old & Naveh-Benjamin, 2008). Thus far, the majority of research on associative memory has employed nonsocial stimuli, despite the fact that much of the information in our daily lives is social. Building on recent research investigating how social content and encoding strategies can affect associative memory performance in younger and older adults (Chang et al., 2025), the current study investigates cross-cultural differences in these processes.

Changes in socioemotional priorities with age could impact associative memory for social information. For example, older adults prioritize emotionally gratifying information and the relationships that are most meaningful to them (Carstensen et al., 1999), which can support cognitive health (Charles & Carstensen, 2010), potentially reflecting efforts to compensate for age-related cognitive declines. In turn, limitations in cognitive resources may cause older adults to be more selective in memory, such as attending less to information they consider unimportant which leads to forgetting (Castel et al., 2011). In further support of this account, older adults’ memory for impressions (Cassidy & Gutchess, 2012) and associations (Hargis & Castel, 2017) improves when they encode this information in a socially meaningful way. These findings suggest that associative deficits may be minimized for social (vs nonsocial) information, which may align with older adults’ socioemotional goals.

This idea was recently tested by Chang et al. (2025), who further recognized that memory paradigms with older adults have tended to incorporate simple social stimuli (e.g., human faces), rather than focusing on more complex social information related to meaningful *interactions*. In addition, prior studies investigating associative memory for meaningful information (Cassidy & Gutchess, 2012; Hargis & Castel, 2017) tended to invoke some level of self-involvement when participants were asked to encode the stimuli in socially relevant ways, but this factor was not explicitly manipulated or separated from socially-relevant information. Thus, it is possible that these mnemonic benefits from social information were actually driven by self-related thought. Indeed, self-referential encoding (i.e., “does this describe you?”) has been found to support item memory (Symons & Johnson, 1997) over structural, phonemic, and semantic encoding (Rogers et al., 1977). Relating information to oneself is also more effective than relating information to a distant other, known as the self-reference effect in memory (SRE). Studies with Western samples have found support for a robust SRE for younger and older adults (Glisky & Marquine, 2009; Gutchess et al., 2007; Hamami et al., 2011; Leshikar et al., 2015; Mueller et al., 1986). Thus, studying meaningful social stimuli in conjunction with manipulating a self-referencing strategy would allow us to tease apart the unique effects of each on memory, and examine whether the mnemonic benefits they produce across age are additive. The recent Chang et al. (2025) study tested this, finding that self-referencing tended to benefit associative memory over other-referencing when scenes contained no or low levels of social content (i.e., limited information related to meaningful social interactions). However, when scenes contained high levels of social content, other-referencing was in fact more beneficial than self-referencing for older adults; for younger adults, performance did not differ between the self- and other-reference conditions.

The somewhat unexpected pattern of results in Chang et al. (2025), in which self-referencing was less effective than other-referencing for high social content, could reflect the contribution of cultural background. Their results are based on a Taiwanese sample, whereas most of the self-referencing literature as well as the few studies investigating the influence of socially meaningful processes on associative memory (e.g., Cassidy & Gutchess, 2012; Hargis & Castel, 2017) are based on Western (e.g., American) samples. The ways in which individuals conceptualize of the self are shaped by cultural context (Markus & Kitayama, 1991). Collectivistic cultures (e.g., Taiwan; China) tend to promote an interdependent self-construal (Markus & Kitayama, 1991), whereas individualistic cultures (e.g., the United States; UK) prioritize an independent self-construal (Markus & Kitayama, 1991). In terms of self-referencing effects in memory, there is some evidence that thinking about a close other boosts Easterners’ memory over self-referencing (Zhu & Zhang, 2002; Zhu et al., 2007). To our knowledge, only one study (Zhang et al., 2020) has explicitly compared self-referencing in younger and older adults across cultures (Taiwanese vs. American). Consistent with past research, self-referencing similarly benefited both American younger adults and older adults’ memory. Contrary to hypotheses and prior findings with young adults (Zhu & Zhang, 2002; Zhu et al., 2007), younger Taiwanese exhibited an SRE of a similar magnitude to that of younger Americans, compared to a close-other condition. Taiwanese older adults, in contrast, had a reduced SRE.

Therefore, to further understand the ways in which social content and self-referencing jointly affect associative memory, we sought to extend the results of Chang et al. (2025) across cultures, allowing us to identify cultural differences in these processes, or, alternatively, find support for the universality of these effects. This study also provided the opportunity to replicate the pattern of findings from Chang et al. (2025) in new samples of Taiwanese younger and older adults, given the novelty and unexpected findings in their study.

Thus, the present study investigates self-referencing in the context of older adults’ socioemotional priorities, as these may differ across cultures. In addition to the evidence for cross-cultural differences in self/other-referencing in memory, recognition of socially salient object-scene pairs also differs across cultures, with East Asian younger and older adults performing better than North American younger and older adults (Yang et al., 2013). The current study builds on these findings by studying how the interaction between self-referencing and the degree of social content impacts associative memory in younger and older adults from the US and Taiwan.

## Hypotheses

Focusing on the new contributions of the present study in terms of culture, we predict a four-way interaction between age, culture, reference condition, and social information on associative memory. Chang et al. (2025) found that Taiwanese older adults’ memory benefited more from other-referencing (vs. self-referencing) for high social information. In contrast, Taiwanese younger adults’ memory for high social information was similarly affected by self- or other-referencing. We predict that we will replicate this pattern in a new sample of Taiwanese participants. The primary aim of this study, however, is to assess cultural differences. (see Table 1 for summary). We hypothesize that self-referencing will benefit Americans’ memory more than other-referencing and that this effect will be stronger with age for more social information; this pattern will emerge in a three-way interaction in the American sample as we break down the overall four-way interaction. Moreover, for American older adults, we predict that a two-way interaction will emerge such that self-referencing benefits memory over other-referencing for more social information, in line with age differences in socioemotional goals. However, the difference between self-referencing and other-referencing will be less pronounced in American younger adults for more social information, suggesting that self/other-referencing encoding strategies do not affect the memorability of socioemotional content.

These predictions are informed by three key mechanisms. First, we reason that older adults’ greater accrual of experience in their respective cultures is reflected in stronger information processing strategies and biases (Gutchess & Cho, 2024; Park et al., 1999). Second, cognitive declines with age may limit older adults’ ability to employ mnemonic strategies that contradict their cultural beliefs and values (Zhang et al., 2020). It is possible that Taiwanese older adults, whose culture values interdependent self-construal, will have a harder time remembering information when relating it to the self. The opposite could be true for American older adults, whose culture endorses an independent self-construal, making self-referencing a more salient strategy. Third, because high-value and social (vs. nonsocial) information tends to have larger effects on memory for older than younger adults (Cassidy & Gutchess, 2012; Hargis & Castel, 2017), it is possible that older adults will remember this information relatively better than young, with older Americans deriving greater value from highly social information when it is related to the self, compared to older Taiwanese. That is, because Taiwanese culture places relatively less value on the self, the mnemonic effects of self and social information will not be additive for them. Thus, the present study advances our understanding of cultural influences on cognitive aging as well as self-referencing and the associative deficit hypothesis (Naveh-Benjamin, 2000) by distinguishing between different levels of social information (Chang et al., 2025).

## Methods

### Participants

Data was collected from the Taiwanese sample between 25 July 2023 and 28 August 2025, and between 20 September 2023 and 18 July 2025 for the American sample. Final samples consisted of 36 participants per group. Initially, we matched the sample size for the Americans to that of the Taiwanese sample reported in Chang et al. (2025), using their power analysis to justify samples of 25 per group to detect a three-way interaction. Based on initial feedback from reviewers, we then increased our sample size to 36 for each group after exclusions. This corresponded to running an additional counterbalancing sequence (i.e., for a total of 3 participants from each age group for each of the 12 counterbalanced versions of the task). Finally, to benefit future work, we conducted a sensitivity analysis using G* Power 3.1. With a total sample size of *N* = 144, an *α* of .05, and power of .80, the study was powered to detect a minimum effect size of *η*_*p*_^2^ = .009. Additional excluded participants were five American younger adults (*n* = 3 for errors in data collection; *n* = 1 for failure to meet the eligibility criteria described below; *n* = 1 for a high score [of 33] on the Beck Depression Inventory-II) and five American older adults (*n* = 2 for an error in data collection; *n* = 1 for a low score [of 23] on the Mini Mental State Exam; *n* = 2 high score [of 7 and 8, respectively] on the Geriatric Depression Scale – Short Form).

American younger adults were undergraduates at Brandeis University and received course credit in exchange for their participation. American older adults were recruited from the community and paid for their participation. American participants originated from the United States, had not lived outside of the US for more than 2 years in a non-Western country, and did not identify as Asian American. Taiwanese participants were recruited from Taipei and paid for their participation. They were originally from Taiwan and verified that their native language was Mandarin. We recruited subjects who were aged between 18–31 (younger adults) and 60–82 (older adults), and who lacked of a history of substance abuse or head injury resulting in a loss of consciousness for more than 10 minutes. Older adults were also screened for psychiatric conditions (e.g., depression, anxiety, bipolar, schizophrenia) and neurological conditions (e.g., epilepsy, Alzheimer’s, or Parkinson’s disease). This study was performed in line with the principles of the Declaration of Helsinki. Approval was granted by the Institutional Review Boards of Brandeis University and National Taiwan University for the American and Taiwanese samples, respectively. All participants provided written informed consent. The demographic characteristics are displayed in Table 2.

### Measures and Materials

#### Mood assessment

Given evidence of episodic memory disruptions in depressed patients (Dillon & Pizzagalli, 2018), we assessed participants’ self-reported mood. Younger adults completed the Beck Depression Inventory-II (BDI-II; Beck et al., 1996), consisting of 21 items with participants selecting a response on a scale of 0 (e.g., “I do not feel sad”) to 3 (e.g., “I am so sad or unhappy that I can’t stand it”). A translated version (Wang et al., 2011) was administered to the sample of Taiwanese younger adults. Both versions have good reliability, with α coefficients of 0.85 (Yang et al., 2012) and 0.90 (Osman et al., 1997). To assess the mood of older adults, we administered the Geriatric Depression Scale – Short Form (GDS – SF; Burke et al., 1991), with translated versions provided to Taiwanese older adults (C, 1996). The GDS-SF consists of 15 items (e.g., “are you basically satisfied with your life?”) answered using a dichotomous scale (“yes” or “no”). It has been validated in both Chinese and American samples, with an α coefficient of 0.75 (Zhang et al., 2022). Questionnaires were completed electronically for the American sample and on paper for the Taiwanese sample. Results from the BDI-II and GDS-SF for American and Taiwanese subjects are reported in Table 3. Average scores for each group fell within the normal range.

#### Neuropsychological assessment

To screen for cognitive orientation in the older adult samples, we administered the Mini Mental State Exam (MMSE; Folstein et al., 1975), using a cutoff score of 26. Thus, all older adults scored 26 or above. A translated version was administered to Taiwanese participants (Guo et al., 1988; Shyu & Yip, 2001). Questions were identical across cultures, but Taiwanese participants received modified versions, when applicable, to replace those that only pertain to the American context (e.g., “what county are we in?” was replaced with “what street are we on?”). Results from the MMSE for American and Taiwanese older adults are reported in Table 3. Average scores for each group fell within the normal range.

#### Memory task stimuli

**Figure 1.**
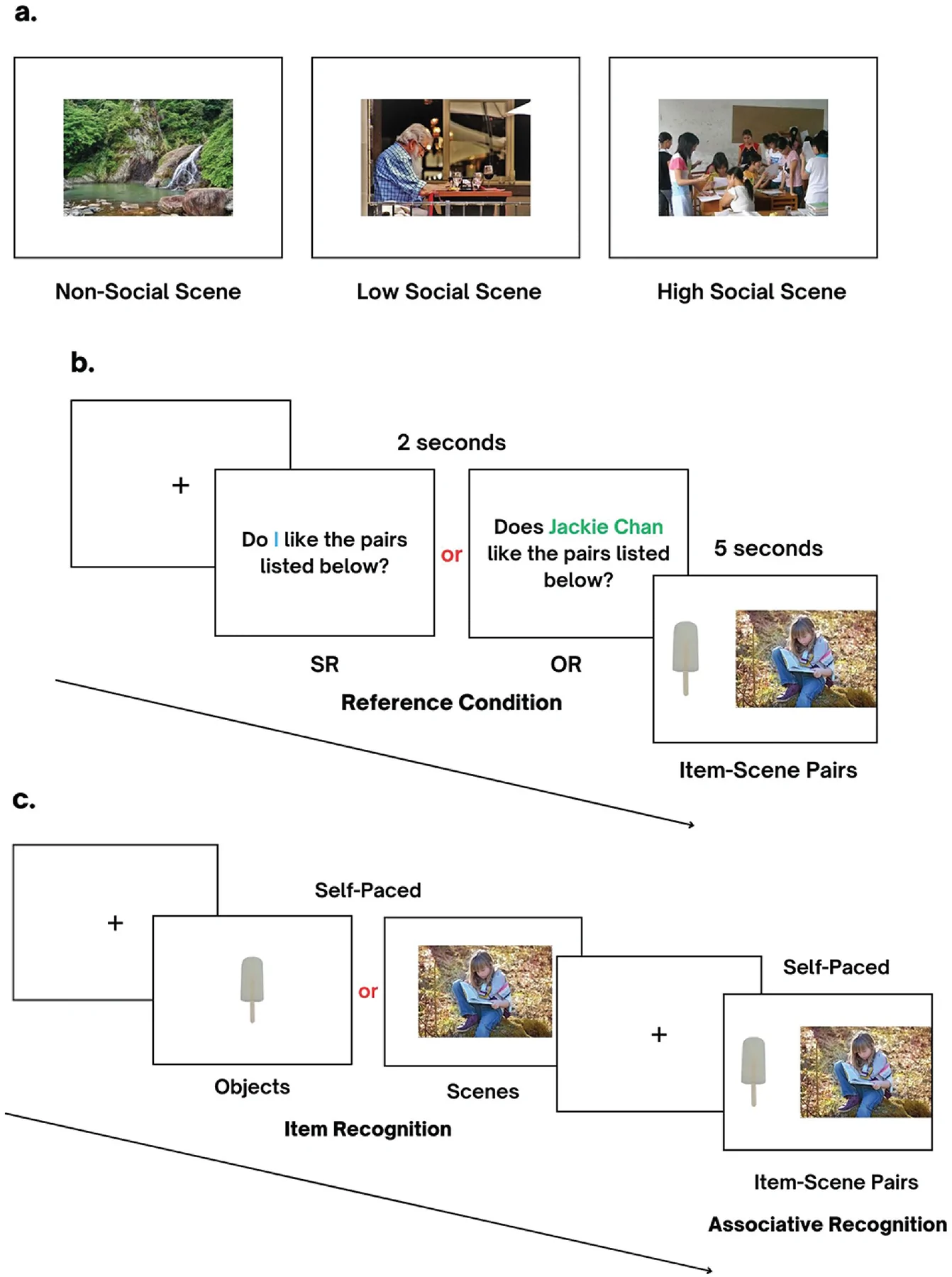
Task stimuli and task schematics.

An object-scene association memory task, based on previous paradigms using inter-item connections (Gutchess & Park, 2009), was used to assess individuals’ associative memory. The task consisted of two phases: a study phase and a test phase, with 96 images of object-scene pairs in total. Six of these were used for the practice trial, the other 60 were used as targets during the study phase, and the final 30 were added as new items to the test phase. The stimuli were further divided into three study-test blocks. Each study phase consisted of 20 trials in which participants related the images to either themselves or a public figure (Jackie Chan; see next section). Each test phase consisted of an item recognition test (i.e., asking whether an object or scene was displayed during the study phase or not) and an associative recognition test (i.e., asking whether the *pair* was displayed during the study phase or not). In the item recognition test, there were 40 trials per block, including 20 old items and 20 new items (i.e., 10 objects and 10 scenes in each). Similarly, the associative recognition test contained 20 trials per block, including 10 intact pairs from the study phase and 10 recombined pairs that contained the same social information as the original image. Items appearing in the item recognition test could also appear in the associative recognition test. As shown in Figure 1(a), the items displayed everyday objects (e.g., an apple), while the scenes differed in the amount of people they portrayed: nonsocial (NS; containing no people), low social (LS; containing one person), and high social (HS; containing at least two people interacting and engaged in a collective activity, like playing chess). There were an equal number of images per social information type (20 each), and all the stimuli were displayed in color. Images were sourced from a variety of free and paid (e.g., Shutterstock) online databases. A separate sample of Taiwanese younger adults rated the images for familiarity, valence, visual complexity, and the extent to which the image depicted an interpersonal interaction (see Chang et al., 2025).

#### Jackie Chan familiarity

Because Jackie Chan served as the other-referencing target in Taiwan in the original Chang et al. (2025) paper, the basis for this study, we administered questions to Americans to ensure that Jackie Chan was also a salient other-referencing target. First, participants were verbally asked, “do you know who Jackie Chan is?” If the subject just said “yes” or “maybe,” they were prompted further, “could you please give me a short description of who Jackie Chan is?” until they provided a response that encompassed his identity as an actor and martial artist. If such a response was provided, participants were subsequently told, “correct - that is all you will need to know about him for this study. Do you feel confident in your response, or would you like even more information?” If participants did not know who Jackie Chan was, answered incorrectly, or wanted more information, they were shown a picture of him with the following question: “This is an image of Jackie Chan. Do you know who he is now, or would you like more information?” Participants who wanted to know more were given a short summary of Jackie Chan with the instructions to read it at their own pace and let the experimenter know when they were done. The level of requested information (1 = immediately knew who Jackie Chan was; 2 = needed printed image; 3 = needed paragraph summary) was recorded. Next, participants answered the following question prior to the demographic questionnaire: “How familiar (1–7) are you with Jackie Chan? One means that you are entirely unfamiliar/don’t even recognize the name, and 7 means that you have a great deal of familiarity and knowledge about him.” Participants responded to a 7-point Likert scale ranging from “very unfamiliar” to “very familiar”; see Supplemental Materials for results.

### Procedure

Upon their arrival, participants signed an informed consent form notifying them about the general purpose of the experiment. Next, they completed the first check of their familiarity with Jackie Chan (“do you know who Jackie Chan is?”). The experimenter then directed them to an online questionnaire (~10 minutes), in which they answered the second question about Jackie Chan (“how familiar [1–7] are you with Jackie Chan?”). Participants next completed the demographic and mood questionnaires (i.e., BDI-II for younger adults and GDS-SF for older adults). Older adults subsequently completed the MMSE (~10 minutes). All tasks were administered in English for American and Mandarin for Taiwanese participants.

The remainder of the experiment was devoted to the associative memory task (~35 minutes), which was separated into three study-test blocks. The study-test blocks were split into three parts to allow for breaks and to make the task more manageable by reducing the memory load. To counterbalance the orders of the stimuli across participants, Chang et al. (2025) created two versions of the task with the same stimuli; the order of presentation was counterbalanced within each version across participants. Samples were tested using E-Prime 2.0 (Taiwanese) and 3.0 (Americans) (Psychology Software Tools, Pittsburgh, PA).

To begin the associative memory task, subjects completed a practice trial consisting of study, distraction, and test phases (see Figures 1(b–c)). During the study phase, they were instructed to relate the stimuli to either themselves (SR; self-referencing) or to Jackie Chan (OR; other-referencing). These instructions were presented for 2 seconds, and were based on perceived like or dislike: “do I like the pairs listed below?” (SR), or “does Jackie Chan like the pairs listed below?” (OR). The image pairs were then displayed for 5 seconds, and participants responded during this period by pressing the p key (labeled as Y, meaning “yes”) or q key (labeled as N, meaning “no”). Because repeatedly shifting between SR and OR may be cognitively taxing or prevent participants from fully engaging with the reference condition, the study phase was divided into four blocks of five trials, two blocks per reference condition. An inter-stimulus interval of 500 milliseconds preceded each trial (see Figure 1(b)).

Before the test phase, a distraction task (~30 seconds) was presented for 10 trials, which involved deciding which number (among two options) on a screen was larger, using the same labeled keys as before. The test phase was divided into two test parts: item recognition and associative recognition; both tasks were self-paced. For item recognition, participants viewed an object or a scene and used the labeled keys to indicate whether they have seen the image before or not; see Supplemental Materials for item memory results. The same was true for the subsequent associative recognition test, except participants judged whether the pairs were intact (e.g., whether the object and scene were paired together at the study phase) or recombined (see Figure 1(c)). Once complete, these procedures were repeated for the remaining two study-test blocks. After completion of all three blocks, participants were thanked, paid, and debriefed.

### Memory performance scores

Participants’ hit rates (i.e., the proportion of old trials that were correctly identified as targets) and false alarm rates (i.e., the proportion of new trials that were incorrectly identified as targets) were converted to discriminability (*d’*; Stanislaw & Todorov, 1999) scores in Excel using the normal inverse cumulative distribution function: NORMINV(hit rate,0,1) – NORMINV(false alarm rate,0,1). To calculate *d’* scores, we matched the procedure of (Chang et al., 2025) and converted hit rates of 1.00 and false alarm rates of .00 to .90 and .10, respectively (Macmillan & Kaplan, 1985).

## Results

Violations of assumptions of sphericity and homogeneity of variance are noted and corrected for in analyses.

### Associative memory performance

#### ANOVA of age, culture, reference condition, and social information on associative memory performance

To compare associative memory performance, we performed a 2 (age: older and younger adults) × 2 (culture: American and Taiwanese) × 2 (reference condition: self and other) × 3 (social content: none [NS], low [LS], high [HS]) mixed ANOVA. Our between-subjects factors were age and culture and our within-subject factors were reference condition and social information. Focusing on the critical four-way interaction of age, culture, reference condition, and social information, we found a significant interaction, *F*(2, 280) = 3.15, *p* = .04; *η*_*p*_^2^ = .02. For completeness, we report associative memory hits and false alarms by condition in the Supplemental Materials. All other results are reported in Table 4.

#### Follow-up ANOVAs of culture, reference condition, and social information on associative memory within each cultural group

To further understand the nature of the four-way interaction, we separately analyzed data for Americans and Taiwanese by performing a 2 (age: older and younger adults) × 2 (reference condition: self and other) × 3 (social content: none [NS], low [LS], high [HS]) mixed ANOVA for each cultural group. This approach also allows us to assess whether we replicated the pattern of results from Chang et al. (2025) in these new Taiwanese samples.

**Figure 2.**
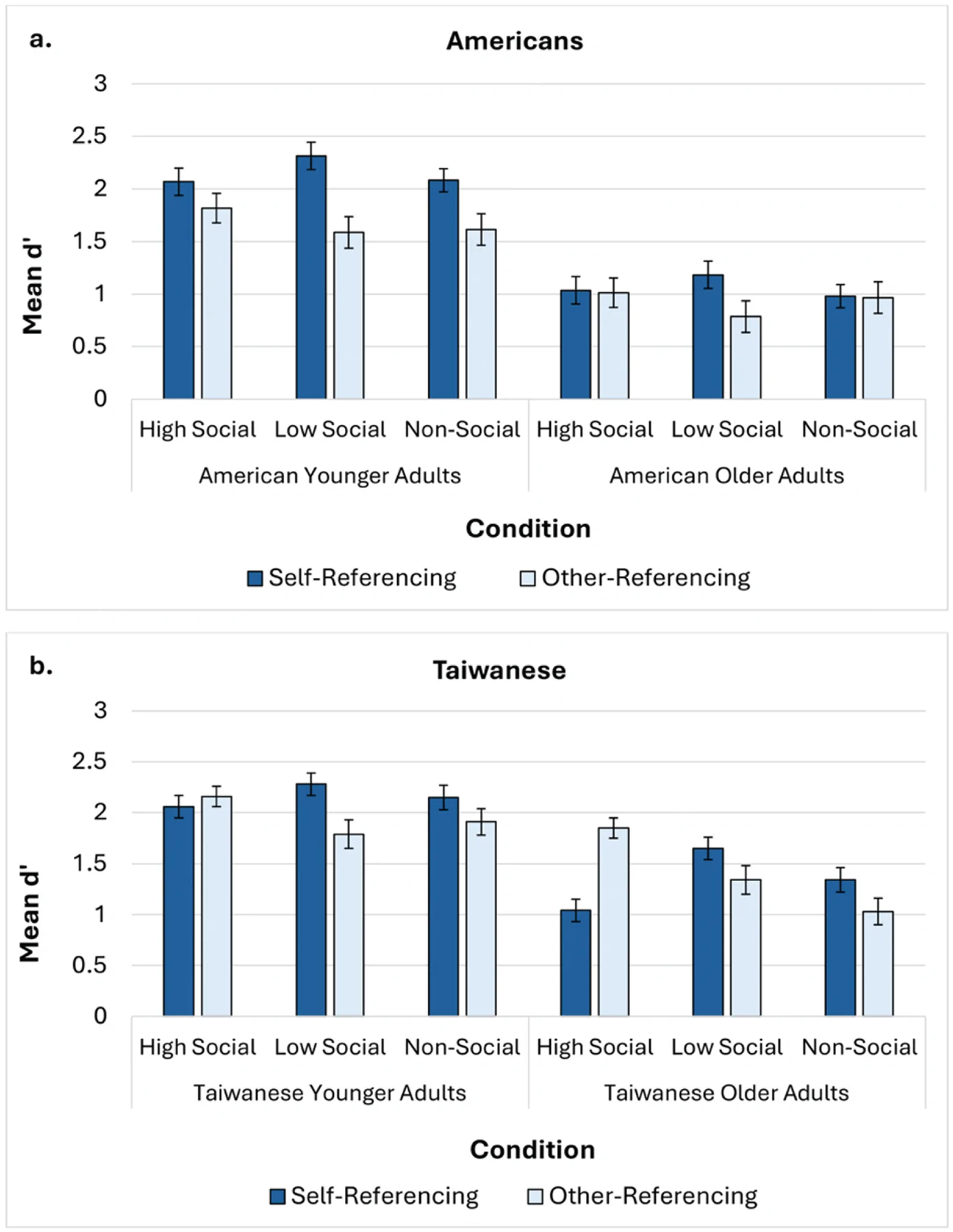
The influence of age, reference condition, and social information on associative memory (d’) in Americans and Taiwanese. Error bars represent between-subject standard errors.

For Americans, the critical three-way interaction of age, reference condition, and social information was not significant, *F*(2, 140) = .31, *p* = .74; *η*_*p*_^2^ = .004. Results are displayed in Figure 2(a). For Taiwanese, the critical three-way interaction of age, reference condition, and social information, was significant, *F*(2, 140) = 4.02, *p* = .02; *η*_*p*_^2^ = .05. Results are displayed in Figure 2(b). Full results for follow-up analyses are available in the Supplemental Materials.

#### Follow-up ANOVAs of reference condition and social information on associative memory within each cultural group

Next, we sought to understand whether the two-way interaction between reference condition and social information differed across age within each culture. This approach aligns with that reported in Chang et al. (2025), allowing us to determine whether their results replicate in a new sample of Taiwanese participants and to directly test our hypothesis that the combination of reference condition and social information would impact American younger and older adults differently. Thus, we separately analyzed the data by performing a 2 (reference condition: self and other) × 3 (social content: none [NS], low [LS], high [HS]) repeated measures ANOVA within each group.

For American younger adults, we found a significant interaction between reference condition and social information, *F*(2, 70) = 3.25, *p* = .045; *η*_*p*_^2^ = .09. Paired samples t-tests were used to clarify the nature of this interaction within each social condition in younger Americans. We found that American younger adults performed better with self-referencing than other-referencing on low social trials, *t*(35) = 4.82, *p* = <.001; *d* = .80, and nonsocial trials, *t*(35) = 2.82, *p* = .008; *d* = .47. However, there was no significant difference between self- and other-referencing on performance for high social trials, *t*(35) = 1.75, *p* = .09; *d* = .29. For American older adults, we did not find a significant interaction between reference condition and social information, *F*(2, 70) = 1.95, *p* = .15; *η*_*p*_^2^ = .05.

For Taiwanese younger adults, we found a significant interaction between reference condition and social information, *F*(2, 70) = 5.90, *p* = .004; *η*_*p*_^2^ = .14. Paired samples t-tests were used to clarify the nature of this interaction within each social condition in younger Taiwanese. We found that Taiwanese younger adults performed better with self-referencing than other-referencing on low social trials, *t*(35) = 3.57, *p* = .001; *d* = .60. However, there was no significant difference between self- and other-referencing on performance for nonsocial trials, *t*(35) = 1.93, *p* = .06; *d* = .32, nor high social trials, *t*(35) = .81, *p* = .42; *d* = .14.

For Taiwanese older adults, we found a significant interaction between reference condition and social information, *F*(2, 70) = 17.05, *p* < .001; *η*_*p*_^2^ = .33. Paired samples t-tests were used to clarify the nature of this interaction within each social condition in older Taiwanese. We found that Taiwanese older adults performed better with other-referencing than self-referencing on high social trials, *t*(35) = 6.28, *p* = <.001; *d* = 1.05. But there was no significant difference between self- and other-referencing on performance for low social trials, *t*(35) = 1.84, *p* = .07; *d* = .31, nor nonsocial trials, *t*(35) = 1.77, *p* = .09; *d* = .30.

#### The influence of culture and reference condition on associative memory for high social and low social trials in Taiwanese

These above results suggest that other-referencing benefits memory for high social trials over self-referencing in older Taiwanese, whereas self-referencing benefits memory for low social trials over other-referencing in younger Taiwanese. Thus, to compare the patterns more directly for younger and older Taiwanese, we focused on the high and low social conditions. We performed separate two-way repeated measures ANOVAs comparing performance between reference conditions (self and other) for high social and low social trials with age as our between-subjects factor. Consequently, we found no significant interaction between age and reference condition on performance for low social trials, *F*(1,70) = .61, *p* = .44; *η*_*p*_^2^ = .01. However, a significant interaction was present between age and reference condition on performance for high social trials, *F*(1,70) = 16.59, *p* < .001; *η*_*p*_^2^ = .19. This pattern is consistent with that reported for a separate set of participants in Chang et al. (2025).

## Discussion

The present study examined cultural differences in the ways in which self-referencing and social content interact to potentially support memory with age. Social information was expected to interact with reference conditions in diverging ways across cultures (Yang et al., 2013), reflecting self-construals that focus more on the self (for Americans) or others (for Taiwanese) (Markus & Kitayama, 1991). Specifically, we predicted that for older Taiwanese, these effects of social information would be larger under other-referencing conditions whereas for older Americans, self-referencing would exaggerate the effects of social information.

Our findings replicate Chang et al.’s (2025) results. Older, relative to younger, Taiwanese participants showed higher memory performance for high social information using other-referencing. This supports our hypothesis that the interaction between culture, reference condition and social information will be magnified with age. However, this was only true for the Taiwanese sample. Despite our predictions that American older adults would benefit more from self-referencing as the level of social information increased, older American participants’ memory performance was relatively consistent across self/other-referencing and level of social information. Thus, the critical finding is that the difference between self- and other-referencing was significant for older Taiwanese participants but not significant for older Americans.

In terms of the implications of our results, the finding that Taiwanese older adults were sensitive to the level of social information in the associative memory paradigm, whereas older American subjects were not, points to a cultural difference in cognition. Although collectivism and individualism were not directly measured here, Taiwanese, who are from a more collectivistic culture and generally endorse an interdependent view of the self, may prioritize social information more in memory because it aligns with the goal of maintaining harmony with other people (Markus & Kitayama, 1991). Indeed, memory for goal-relevant information tends to exceed that for goal-irrelevant information (Siegel & Castel, 2018). By contrast, American participants are from an individualistic culture, and view the self as individual and separate from others, perhaps making them less inclined to remember social information (Markus & Kitayama, 1991).

With respect to encoding conditions, our finding that Taiwanese older adults, relative to the other groups, benefited most from high social content in the other-referencing condition provides support for the idea that cultural differences in cognition are amplified with age (Zhang et al., 2020). Because older adults have more experience in their collectivistic culture, they may be more inclined to relate information that is highly distant from the self to another person. As noted by Chang et al. (2025), processing of high social information and engaging in other-referencing (which is similar to mentalizing) may rely on shared brain structures that Taiwanese older adults may be more apt at engaging. We propose that this is due to cultural experience – Taiwanese older adults may be better at assessing the social scenes for the degree of social information in them because they are more attuned to interpersonal dynamics, thus better remembering this information when it is related to another. To this end, it would be interesting to supplement these findings with brain imaging data and to examine whether Taiwanese older adults are more likely to spontaneously engage in other-referential encoding for high social information than Taiwanese younger adults, even when not prompted to do so.

Interestingly, we did not find self-referencing to be a more effective strategy for American older adults, despite literature documenting its efficacy in older Western samples (Glisky & Marquine, 2009; Gutchess et al., 2007; Hamami et al., 2011; Leshikar et al., 2015; Mueller et al., 1986), and findings that self-reference effects in memory were stronger for older Americans than for older Taiwanese (Zhang et al., 2020). It is possible that the combination of social information with self-referencing, the amount of information to be studied, or the demands of an associative memory task mitigate the effectiveness of a self-referencing strategy. Prior literature suggests that older Easterners have an advantage compared to older Westerners in remembering socially meaningful items and their contexts, a type of associative memory (Yang et al., 2013). Our findings support the argument that the presence of social information may be of critical importance in determining cultural influences on memory with age. It may be the case that the effects of culture on both self-referencing and associative memory with age differ dramatically depending on whether the stimuli are social in nature. Thus, more research is needed that investigates the influences of social information on memory across age and culture.

Another possible explanation is that the nature of the paradigm made it difficult for participants to meaningfully relate the images to the self. Prior studies on self-referencing have asked participants to think, “does this describe you?” when exposed to adjectives (e.g., Symons & Johnson, 1997). This prompt may be more meaningful, or encourage participants to engage more deeply with self-referencing than the question we used (i.e., “do I like the pairs listed below?”). In a similar way, the participants may have found the stimuli difficult to relate to the self, causing them to make arbitrary selections when asked to think about their preferences. It is also possible that the scenes were less familiar or that the degrees of sociality were less distinct for Americans. Because the task was created in an East Asian context, it is possible that Americans had a harder time associating stimuli with the self or that the sociality dimension was weaker than for the Taiwanese. If cultural differences in cognition are most apparent in older adults, as our findings seem to indicate, then American older adults would be especially sensitive to the cultural incongruity of the stimuli. Future work should assess the familiarity of these stimuli across cultures, ensuring that there is an equal amount of culturally fair items and scenes.

Other directions for future research would be to test the generalizability of findings under other conditions. For example, it is difficult to discern how this paradigm translates into everyday life since we used static images, as has been the norm for this area of research (e.g., Hargis & Castel, 2017). This study is strong in that it includes complex scenes of varying levels of social information. However, participants’ ability to remember two static images (e.g., an orange and a group of people interacting) may not translate well into everyday life. To increase ecological validity, future work could examine this using more dynamic stimuli, perhaps video clips or narratives, that more closely model naturalistic situations. This study also employed multiple study-test blocks to support levels of performance for a challenging associative memory task. Whether the pattern would replicate for a surprise memory test (i.e., a single retrieval block) is a question for future research. Using Jackie Chan as an other-reference target was initially necessitated by the Chang et al. (2025) paradigm, and we administered additional measures to assure the appropriateness of this target for American samples. Although our American sample reported being familiar with him, and our procedures ensured that they were, future research could use other targets to be certain that particular identities do not contribute to cultural differences. Finally, our samples differed in their years of education. When considering this variable in a cross-cultural context, it is important to acknowledge historical differences in access to education. As discussed in Zhang et al. (2020), older cohorts in the US historically had more access to higher education than in Taiwan (see Gutchess & Boduroglu, 2019 for a discussion of comparing education across cultures); even compulsory education expanded in Taiwan in 1968 and again in 2014. Consistent with these ideas, in a larger study comparing samples collected across these same sites (e.g., Cho et al., 2025), samples of American and Taiwanese older adults were well-matched on neuropsychological measures, despite differences in educational attainment.

Taken together, our findings reveal that mnemonic strategies, such as self-referencing and social information, differ in their effectiveness across ages and cultures. Consequently, recommendations for alleviating age-related memory deficits should acknowledge the ways in which the self and socio-cultural context interact to shape cognitive processing. Conducting research across cultures is important in order to make claims about the universality of findings regarding cognitive aging. So far, samples are relatively homogenous in psychology research, with WEIRD (Western, Educated, Industrialized, Rich, or Democratic) or Global North individuals overrepresented, thereby limiting the applicability of existing theories (Gutchess & Cho, 2024; Gutchess & Rajaram, 2023; Henrich et al., 2010). Here, we discuss our findings in the context of self-construal differences between East Asian and North American cultures (i.e., independence vs interdependence), but we also recognize that other sociocultural variables (e.g., religious heritage) could impact cognition in different contexts, beyond the East–West dichotomy (Kitayama & Salvador, 2024; Vignoles et al., 2016). To successfully move past WEIRD sampling practices, future work should focus on younger and older adults in the Middle East, South Asia, South America, and Africa, in addition to probing nuanced within-country variations in cognition due to factors like biculturalism. Thus, more research is needed to explore the ways in which social information and diverse contexts may impact memory, particularly associative memory, with age.

## Supplementary Material

Supp 1

Supplemental data for this article can be accessed online at https://doi.org/10.1080/13825585.2026.2691113

## Figures and Tables

**Table 1.** Summary of predictions.

Summary of Predictions	
Predicted 4-way interaction between age, culture, reference condition, and social information.	
Prediction for Taiwanese Older Adults (replication of Chang et al., 2025):	Older adults’ memory will benefit more from *other-referencing* (vs. self-referencing) for high social information.
Prediction for Taiwanese Younger Adults (replication of Chang et al., 2025):	Younger adults' memory for high social information will be similarly affected by self- or other-referencing.
Prediction for Americans:	3-way interaction, with *self-referencing* benefiting Americans' memory more than other-referencing. This effect will be stronger with age for more social information.
Prediction for American Older Adults:	2-way interaction, with *self-referencing* benefiting memory over other-referencing for more social information.
Prediction for American Younger Adults:	Difference between self- and other-referencing will be less pronounced for more social information.

**Table 2.** American and Taiwanese Sociodemographic Characteristics.

	American Younger Adults (*n* = 36)	American Older Adults (*n* = 36)	Taiwanese Younger Adults (*n* = 36)	Taiwanese Older Adults (*n* = 36)
**Age, *M*(*SD*)** ***	19.94 (1.51)	71.68 (6.29)	23.06 (3.29)	71.31 (4.73)
**Gender**				
Women, *n* (%)	20 (55.60%)	26 (72.20%)	22 (61.10%)	23 (63.90%)
Men, *n* (%)	13 (36.10%)	9 (25.00%)	14 (38.90%)	13 (36.10%)
Non-binary, *n* (%)	3 (8.30%)	1 (2.80%)	0 (0.00%)	0 (0.00%)
**Race**				
White/Caucasian, *n* (%)	28 (77.80%)	34 (94.40%)	0 (0.00%)	0 (0.00%)
Asian, *n* (%)	0 (0.00%)	0 (0.00%)	36(100.00%)	36(100.00%)
Black/African American, *n* (%)	4 (11.10%)	0 (0.00%)	0 (0.00%)	0 (0.00%)
Native Hawaiian/Other Pacific Islander, *n* (%)	1 (2.80%)	0 (0.00%)	0 (0.00%)	0 (0.00%)
Multiracial, *n* (%)	0 (0.00%)	2 (5.60%)	0 (0.00%)	0 (0.00%)
Not specified	3 (8.30%)	0 (0.00%)	0 (0.00%)	0 (0.00%)
**Ethnicity**				
Not Hispanic, *n* (%)	30 (83.30%)	36(100.00%)	36(100.00%)	36(100.00%)
Hispanic, *n* (%)	6 (16.70%)	0 (0.00%)	0 (0.00%)	0 (0.00%)
**Education in years, *M*(*SD*)** ***	13.52 (1.35)	17.00 (2.74)	15.97 (2.89)	14.50 (2.13)

*** *p* < .001 for the cross-cultural comparisons of age for the younger adults and of education for both younger and older adults (note that education also differed when comparing younger and older adults within cultural groups. More specifically, American older adults had more education than their younger counterparts, while the opposite pattern was true for Taiwanese).

**Table 3.** American and Taiwanese performance on Beck Depression Inventory-II (BDI-II), Geriatric Depression Scale - Short Form (GDS-SF), and Mini Mental State Exam (MMSE).

	American Younger Adults (*n* = 36)	American Older Adults (*n* = 36)	Taiwanese Younger Adults (*n* = 36)	Taiwanese Older Adults (*n* = 36)
BDI-II,*M* (*SD*)***	7.72 (5.28)		4.17 (3.58)	
GDS-SF, *M* (*SD*)		.89 (1.19)		1.14 (1.22)
MMSE, *M* (*SD*)		28.94 (1.31)		28.81 (1.12)

*** *p* = .001 for the cross-cultural comparisons of BDI-II in the younger adults.

**Table 4.** Full results of the ANOVA on associative memory (d’) scores for American and Taiwanese younger and older adults.

Effect	df		*F*	*p*	*η*_*p*_^2^
**Age**	1	140	85.10	**<.001**	.38
**Culture**	1	140	9.17	**.003**	.06
**Reference**	1	140	17.89	**<.001**	.11
**Social Information**	2	280	3.68	**.03**	.03
Age × Culture	1	140	1.87	.17	.01
**Culture × Reference**	1	140	6.84	**.01**	.05
**Age × Reference**	1	140	11.24	**.001**	.07
Age × Culture × Reference	1	140	.11	.74	.001
Culture × Social Information	2	280	.68	.51	.005
Age × Social Information	2	280	.64	.53	.005
Age × Culture × Social Information	2	280	2.11	.12	.02
**Reference × Social Information**	2	280	20.78	**<.001**	.13
**Culture × Reference × Social Information**	2	280	5.00	**.007**	.04
Age × Reference × Social Information	2	280	1.03	.36	.007
**Age × Culture × Reference × Social Information**	2	280	3.15	**.04**	.02

## Data Availability

The datasets generated and analyzed during the current study are available from the corresponding author upon reasonable request.
