# Supplementary material for: Cross-cultural differences in social and self-referential memory are magnified with age
Source: Neuropsychol Dev Cogn B Aging Neuropsychol Cogn. Author manuscript; Available in PMC 2026 Jul 5. (PMC13333165; doi:10.1080/13825585.2026.2691113)
Supplement: Supp 1 [file NIHMS2189865-supplement-Supp_1.docx]

**Supplemental Materials**

**Familiarity with the Other Person Target**

To verify that Jackie Chan was an appropriate other-reference target for Americans, participants answered the question, “how familiar (1-7) are you with Jackie Chan? One means that you are entirely unfamiliar/don't even recognize the name, and 7 means that you have a great deal of familiarity and knowledge about him”. The average response for younger Americans was “mostly familiar” (*M* = 6.08, *SD* = 1.00), while the average response for older Americans was “very familiar” (*M* = 6.94, *SD* = 2.35). Younger and older Americans significantly differed in their familiarity ratings, *t*(47.16) = 2.02, *p* = .049, *d* = .48. When offered additional information about Chan, some of the younger (*n* = 16; 44.40%) and most of the older (*n* = 19; 52.80%) Americans requested it, as opposed to none or receiving the photo only. Based on these results, we established that Jackie Chan was an appropriate target for American participants.

**Item Memory Performance**

Although our focus was on associative memory, we also analyzed participants’ item memory to provide a complete report of their performance on the task. To do so, we performed a univariate ANOVA on item accuracy rates with age and culture as between-subjects factors. Because the new items added during the test phase were not previously encoded as "self" or "other", there are not separate false alarm rates for each scene type (i.e., non-social, low social, or high social) and reference conditions (i.e., self or other). For this reason, we report item memory in terms of mean accuracy rates (i.e., the hit rate plus correct rejection rate divided by 2), as calculated in Chang et al. (2025). Data are reported in Supplemental Table 1.

We found a significant main effect of age, *F*(1, 140) = 15.83, *p* < .001; *η_p_*^2^ = .10, and a significant main effect of culture, *F*(1, 140) = 68.22, *p* < .001; *η_p_*^2^ = .33. Specifically, younger participants outperformed their older counterparts across cultures, and Taiwanese participants performed better than Americans overall. However, there was no significant interaction between age and culture on item accuracy, *F*(1, 140) = 1.38, *p* = .24; *η_p_*^2^ = .01. Taken together, these findings suggest that the differences observed in associative memory across both age and culture are likely not due to differences in item memory alone.

**Supplemental Table 1**

*Item Memory Accuracy Scores for American and Taiwanese Younger and Older Adults*

|  | **American Younger Adults**  **(*n* = 36)** | **American Older Adults**  **(*n* = 36)** | **Taiwanese Younger Adults**  **(*n* = 36)** | **Taiwanese Older Adults**  **(*n* = 36)** |
| --- | --- | --- | --- | --- |
| Item Memory Accuracy, *M(SE)* | .89 (.007) | .85 (.007) | .93 (.007) | .92 (.007) |

In addition, Supplemental Tables 2 and 3 report the hits and false alarm rates used to calculate the d’ scores reported in the main text (presented in Figure 2 in the main text).

**Supplemental Table 2**

*Associative Memory Hits M(SD) for American and Taiwanese Younger and Older Adults*

|  | **American Younger Adults**  **(*n* = 36)** | **American Older Adults**  **(*n* = 36)** | **Taiwanese Younger Adults**  **(*n* = 36)** | **Taiwanese Older Adults**  **(*n* = 36)** |
| --- | --- | --- | --- | --- |
|  | Hits | Hits | Hits | Hits |
| **Self-Referencing** |  |  |  |  |
| High Social | .86 (.10) | .79 (.16) | .84 (.10) | .80 (.12) |
| Low Social | .88 (.06) | .82 (.14) | .86 (.08) | .81 (.12) |
| Non-Social | .84 (.09) | .84 (.10) | .86 (.06) | .79 (.16) |
| **Other-Referencing** |  |  |  |  |
| High Social | .85 (.12) | .83 (.12) | .86 (.10) | .87 (.06) |
| Low Social | .76 (.19) | .78 (.16) | .74 (.16) | .77 (.17) |
| Non-Social | .79 (.14) | .80 (.11) | .80 (.14) | .76 (.18) |

**Supplemental Table 3**

*Associative Memory False Alarms M(SD) for American and Taiwanese Younger and Older Adults*

|  | **American Younger Adults**  **(*n* = 36)** | **American Older Adults**  **(*n* = 36)** | **Taiwanese Younger Adults**  **(*n* = 36)** | **Taiwanese Older Adults**  **(*n* = 36)** |
| --- | --- | --- | --- | --- |
|  | False Alarms | False Alarms | False Alarms | False Alarms |
| **Self-Referencing** |  |  |  |  |
| High Social | .20 (.16) | .46 (.27) | .17 (.10) | .46 (.29) |
| Low Social | .14 (.09) | .43 (.31) | .13 (.07) | .26 (.22) |
| Non-Social | .16 (.07) | .52 (.27) | .17 (.11) | .35 (.27) |
| **Other-Referencing** |  |  |  |  |
| High Social | .27 (.19) | .51 (.28) | .18 (.16) | .28 (.19) |
| Low Social | .24 (.17) | .53 (.29) | .16 (.11) | .33 (.27) |
| Non-Social | .26 (.18) | .48 (.26) | .18 (.12) | .41 (.27) |

**Full Results from Three-way ANOVAs in Americans and Taiwanese**

To fully compare Chang et al. (2025) with our new Taiwanese sample and to provide a complete report of results for the American sample, we provide ANOVA tables for the three-way interaction of age, reference condition, and social information within each culture (Supplemental Tables 4 and 5).

**Supplemental Table 4**

*Full Results of the ANOVA on Associative Memory (d’) Scores for American Younger and Older Adults*

| **Effect** | **df** | | ***F*** | ***p*** | ***η_p_*^2^** |
| --- | --- | --- | --- | --- | --- |
| **Age** | 1 | 70 | 45.16 | **< .001** | .39 |
| **Reference** | 1 | 70 | 21.53 | **< .001** | .24 |
| Social Information | 2 | 140 | .65 | .53 | .01 |
| **Age x Reference** | 1 | 70 | 6.24 | **.02** | .08 |
| Age x Social Information | 2 | 140 | .23 | .80 | .003 |
| **Reference x Social Information** | 2 | 140 | 4.68 | **.01** | .06 |
| Age x Reference x Social Information | 2 | 140 | .31 | .74 | .004 |

**Supplemental Table 5**

*Full Results of the ANOVA on Associative Memory (d’) Scores for Taiwanese Younger and Older Adults*

| **Effect** | **df** | | ***F*** | ***p*** | ***η_p_*^2^** |
| --- | --- | --- | --- | --- | --- |
| **Age** | 1 | 70 | 40.74 | **< .001** | .37 |
| Reference | 1 | 70 | 1.43 | .24 | .02 |
| **Social Information** | 2 | 140 | 3.56 | **.03** | .05 |
| **Age x Reference** | 1 | 70 | 5.00 | **.03** | .07 |
| Age x Social Information | 2 | 140 | 2.41 | .09 | .03 |
| **Reference x Social Information** | 2 | 140 | 21.77 | **< .001** | .24 |
| **Age x Reference x Social Information** | 2 | 140 | 4.02 | **.02** | .05 |
